# Multiple Applications of Nanomaterials in the Diagnosis and Treatment of Hemorrhagic Stroke

**DOI:** 10.3390/biom15091272

**Published:** 2025-09-03

**Authors:** Boyao Yuan, Taotao Jiang, Jingjing Han, Ting Zheng, Manxia Wang

**Affiliations:** The Department of Neurology, Lanzhou University Second Hospital, Lanzhou 730030, China; 220220905361@lzu.edu.cn (B.Y.); jiangtt2024@lzu.edu.cn (T.J.); hanq2021@lzu.edu.cn (J.H.); ery_zhengtery@lzu.edu.cn (T.Z.)

**Keywords:** nanotechnology, nanomaterials, hemorrhagic stroke, diagnosis, treatment

## Abstract

Hemorrhagic stroke is a severe cerebrovascular disease with a high rate of disability and mortality. Its complex pathological mechanisms, such as blood–brain barrier damage, neuroinflammation, and oxidative stress, along with the restrictive nature of the blood–brain barrier, have restricted the clinical therapeutic effects of drugs. Nanotechnology, with its advantages of targeting ability, biocompatibility, and multifunctionality, has provided a new approach for the precise diagnosis and treatment of hemorrhagic stroke. In terms of diagnosis, imaging technology enhanced by magnetic nanoparticles can achieve real-time bedside monitoring of hematoma dynamics and cerebral perfusion, significantly improving the timeliness compared with traditional imaging methods. In the field of treatment, the nanodrug delivery system can remarkably improve the bioavailability and brain targeting of clinical drugs and herbal medicines by enhancing drug solubility, crossing the blood–brain barrier, and responsive and targeting drug release. Multifunctional inorganic nanomaterials, such as cerium oxide nanoparticles, graphene, and perfluorooctyl octyl ether nanoparticles, can alleviate brain edema and neuronal damage through antioxidant and anti-inflammatory effects, and the scavenging of free radicals. Moreover, gene delivery mediated by nanocarriers and stem cell transplantation protection strategies have provided innovative solutions for regulating molecular pathways and promoting nerve repair. Although nanotechnology has shown great potential in the diagnosis and treatment of hemorrhagic stroke, its clinical translation still faces challenges such as the evaluation of biosafety, standardization of formulations, and verification of long-term efficacy. In the future, it is necessary to further optimize material design and combine multimodal treatment strategies to promote a substantial breakthrough in this field from basic research to clinical application.

## 1. Introduction

**Figure 1:**
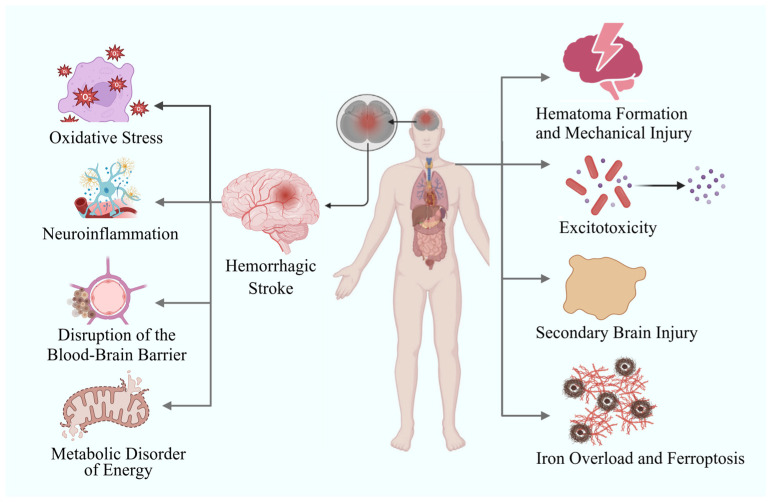
Pathophysiological mechanism and related complications of hemorrhagic stroke.

With the increase in aging and people’s current sub-healthy lifestyle, stroke, as the main clinical type of cerebrovascular diseases, has become the second leading cause of human death. Together with heart disease and malignant tumors, it constitutes the three major diseases that cause death and disability in humans [1,2]. According to statistics, stroke causes 5.5 million deaths globally every year, and half of the survivors are left with complications such as disabilities [1]. Approximately 20% of strokes are hemorrhagic strokes, and 80% are ischemic strokes [3]. In stroke patients, brain cells will suffer continuous and irreversible damage. After hemorrhagic strokes, the extravasation of blood components activates microglia and astrocytes, which release pro-inflammatory factors to trigger neuroinflammation [4] and exacerbate neuronal damage. Meanwhile, hemoglobin degradation products induce the production of a large amount of reactive oxygen species, triggering oxidative stress and damaging the structures of lipids, proteins, and nucleic acids [5,6,7]. The mechanical pressure from blood accumulation, combined with inflammatory mediators, leads to the disruption of tight junctions in the blood–brain barrier [8], increased vascular permeability, and subsequent cerebral edema. In addition, iron ion deposition inhibits mitochondrial respiratory chain function, resulting in reduced ATP production and energy metabolism disorders, which further exacerbate neuronal apoptosis. These pathological processes superimpose on each other, forming secondary post-hemorrhagic injury, expanding the scope of brain damage and worsening neurological deficits (Figure 1). And these pathophysiological mechanisms can further cause other complications, such as post-stroke depression [9], cognitive impairment [10], epilepsy [11], and dysphagia [12].

These symptoms not only reduce the quality of life of stroke patients, causing functional disabilities, and increase the long-term mortality rate, but also increase the economic burden on society and the patients’ families [13]. Hemorrhagic stroke is a serious disease in which the intracranial blood vessels rupture and bleed due to non-traumatic factors, resulting in cerebrovascular nerve dysfunction and even endangering life. In clinical practice, the common types of hemorrhagic stroke are primarily categorized into intracerebral hemorrhage (ICH) and subarachnoid hemorrhage (SAH). Furthermore, due to the complex pathophysiological mechanisms inherent in hemorrhagic stroke—such as neuroinflammation, apoptosis, and vasospasm—patients with cerebral hemorrhage often present with severe complications, including delayed cerebral infarction, cerebral edema, and elevated intracranial pressure [14,15]. Despite significant advances in surgical strategies and intensive care, the overall prognosis for hemorrhagic stroke patients remains suboptimal, manifested by persistently high rates of mortality and disability. This is primarily attributed to early rebleeding and devastating secondary injuries (cerebral edema and inflammation). Moreover, these challenges are compounded by the limitations of current pharmacotherapy—particularly the difficulty in achieving targeted drug delivery across the blood–brain barrier (BBB)—and the lack of effective early-stage interventions for secondary damage [16]. Preclinical studies have demonstrated that neuroprotective agents (compounds that can protect neurons from damage, degeneration, or death caused by various pathological factors) exhibit promising therapeutic effects in stroke management [17]. However, conventional drug administration is constrained by the selective permeability of the BBB, efflux pumps, and the inherently low bioavailability of the drugs themselves [18], which limits the entry of most neuroprotective agents into the brain and compromises their therapeutic efficacy. This underscores the critical need to develop novel strategies to enhance drug delivery to the brain in hemorrhagic stroke patients, with the aim of improving survival rates and post-stroke quality of life.

**Figure 2:**
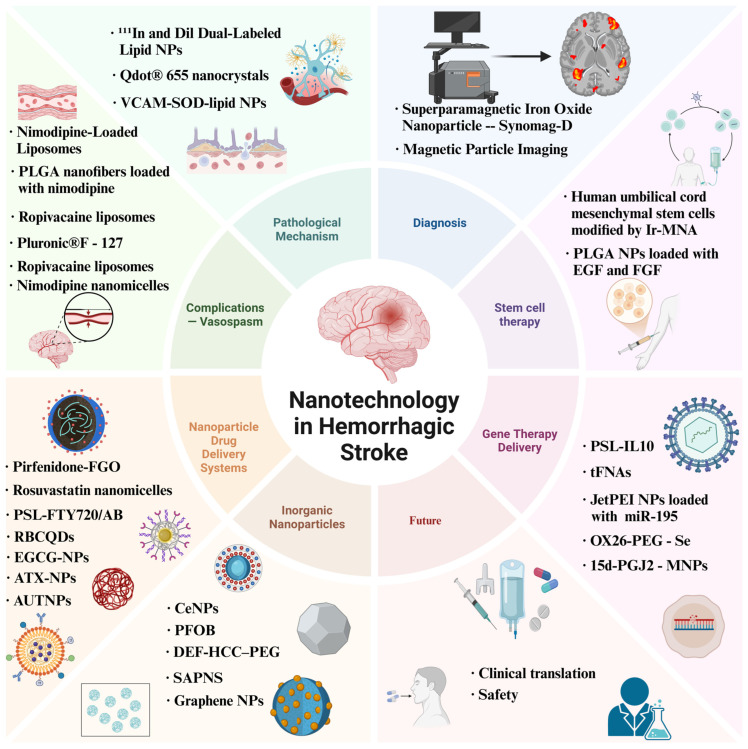
Multiple applications of nanotechnology in the diagnosis and treatment of hemorrhagic stroke.

Nanotechnology represents a cutting-edge technology applied in the medical field, leveraging nanoscale materials and devices to exert significant impacts across various diseases including cancer and stroke [19,20]. The multifunctional biological properties and high biocompatibility inherent in its diverse forms, such as nanoparticles [21], nanodelivery systems [22], and nanozymes [23], coupled with precise targeting at the cellular and molecular levels [24], have pioneered novel avenues for traversing the BBB, facilitating drug transport, and enabling effective therapeutics [25]. As systematically visualized in Figure 2, these multifunctional advances establish nanotechnology as one of the most promising strategies for diagnosis and treatment of hemorrhagic stroke. Therefore, this study innovatively and systematically integrates the research findings of nanotechnology in the field of drug delivery for hemorrhagic stroke. It establishes cross-dimensional connections among studies scattered across different material systems and various modification strategies. Furthermore, it explores and analyzes the application value of nanotechnology in the diagnosis of cerebral hemorrhage. The purpose of this research is to provide a clearer research context for scholars in this field and promote the translational application of nanotechnology in the clinical diagnosis and treatment of hemorrhagic stroke.

## 2. Role of Nanotechnology in Investigating Pathological Mechanisms and Precision Diagnosis of Hemorrhagic Stroke

The BBB, a dynamic neuroprotective interface within the neurovascular unit, comprises brain microvascular endothelial cells interconnected by tight junction proteins, basement membranes, pericytes, astrocytic end-feet, and oligodendrocytes. While endothelial tight junctions form the primary physical barrier [26], oligodendrocytes contribute to BBB integrity through myelination-dependent metabolic coupling and secretion of neurotrophic factors that stabilize cerebral microvasculature. This coordinated cellular network maintains neuronal microenvironment homeostasis by selectively regulating blood-to-brain substance exchange [27].

However, while the BBB’s barrier properties prevent pathogen invasion, they also severely restrict the delivery of therapeutic drugs into the brain. Notably, cerebral hemorrhage induces dynamic changes in BBB integrity, creating a potential temporal window for targeted drug delivery. Thus, understanding the mechanisms and temporal framework of BBB alterations after cerebral hemorrhage is critical for developing novel therapeutic strategies. Using a collagenase-induced striatal ICH model in mice, Al-Ahmady’s team [28] employed radioactive indium (^111^In) and Dil dual-labeled lipid nanoparticle technology to reveal the temporal characteristics of BBB damage: biphasic accumulation peaks occurred at 3–24 h and 48–72 h post-intravenous injection, with intrathecal liposome concentrations increasing by 27.5-fold and 11-fold compared to the control group, respectively. SPECT/CT (Single-Photon Emission Computed Tomography/Computed Tomography) dynamic imaging demonstrated that liposomes accumulated most significantly in the hematoma region when administered 24 h after ICH, with no notable uptake observed in the contralateral brain region or healthy controls. Fluorescence tracing and histological analysis further confirmed that liposomes primarily entered the actively bleeding area through damaged blood vessels and co-localized with activated microglia. Mechanistic studies indicated that early after ICH (3–5 h), Caveolin-1-mediated transcytosis was enhanced, whereas late-stage (48–50 h) tight junction protein Claudin-5 was significantly downregulated, suggesting a dual regulatory mechanism underlying BBB permeability changes. Reyes-Esteves et al. [29] quantified the temporal characteristics of vascular leakage following ICH through chromium-51-labeled red blood cell and albumin tracer experiments. Their findings revealed that red blood cell extravasation significantly decreased within 4 h and essentially disappeared by 24 h, whereas albumin leakage persisted beyond 4 h post-injury. Notably, the brain uptake of vascular endothelial-targeted superoxide dismutase liposomes was significantly higher than that of non-targeted liposomes, and their delivery efficiency remained stable after the acute phase, in contrast to the rapid decline in delivery efficiency via passive leakage pathways. This discrepancy provides critical evidence for optimizing targeted drug delivery strategies. Additionally, in SAH, cortical microcirculation disorder represents a core pathological mechanism leading to global cerebral ischemia. Ishikawa et al. [30] utilized Qdot^®^ 655 nanocrystals combined with in vivo two-photon microscopy to observe that within one minute of SAH, acute changes occurred, including capillary network disruption and an 85% reduction in pre-arteriolar blood flow velocity. Although arterioles triggered autoregulatory dilation, blood flow failed to recover, and the vasodilation response was unsustained, indicating decompensation of microcirculatory autoregulation in the early stage of SAH. This finding establishes a critical time window for ultra-early intervention.

In clinical practice, the diagnosis of hemorrhagic stroke necessitates the integration of clinical symptom assessment, neuroimaging examinations, and laboratory tests, with neuroimaging serving as a pivotal step in establishing a definitive diagnosis. Commonly used neuroimaging modalities include cranial CT and brain magnetic resonance imaging (MRI), which enable rapid detection of the presence and location of hemorrhagic stroke, as well as quantification of hemorrhage volume. In certain cases, clinicians may perform cerebral angiography to evaluate the site and severity of vascular rupture. Research indicates that more than two-thirds of patients experience early neurological deterioration within 48 h due to hematoma expansion; thus, frequent neurological examinations facilitate early identification of signs of clinical deterioration and increased intracranial pressure [31]. While CT and head MRI are highly accurate in detecting intracranial hemorrhage, they are associated with substantial patient costs, exposure to relatively high radiation doses, and contraindications. Thus, the development of a continuous, non-invasive bedside monitoring technique capable of assessing intracranial hemorrhage and cerebral perfusion in these patients would be highly valuable. Magnetic particle imaging (MPI), an emerging biomedical imaging technology, employs superparamagnetic nanoparticles as contrast agents to generate images by detecting their dynamic responses within magnetic fields. This modality enables real-time, non-invasive, and precise cardiovascular imaging while offering excellent contrast, sensitivity, spatial and temporal resolution, safety, and biocompatibility [32]. Szwargulski et al. [33] pioneered the application of MPI technology in ICH detection. By intravenously administering the superparamagnetic iron oxide nanotracer—Synomag-D, they achieved bedside detection in just 3 min, significantly improving timeliness compared to the 11–13 min required for conventional CT/MRI. MPI not only enables clear differentiation between liquid and coagulated regions of hematomas but also allows simultaneous monitoring of cerebral perfusion, providing critical evidence for selecting optimal surgical timing. Notably, during minimally invasive catheter aspiration, MPI can dynamically optimize catheter positioning and monitor hematoma evacuation efficacy in real time. Furthermore, MPI exhibits high sensitivity in identifying complications such as intracranial pressure elevation and vasospasm, facilitating early intervention through targeted therapy and reducing the risk of secondary brain injury. Furthermore, studies indicate that improving the biocompatibility of magnetic particles and MPI tracers primarily relies on the rational selection and application of biocompatible coating agents. For instance, in the preparation of magnetic particles, employing biocompatible coatings such as polyacrylic acid, lauric acid, and malic acid can effectively control nanoparticle size, prevent aggregation, and enhance biocompatibility through their non-toxic properties. For MPI tracers like zinc ferrite nanoparticles, these coatings further optimize structural morphology and magnetic properties, laying the foundation for clinical applications. Additionally, using sodium carboxymethyl dextran as a coating material ensures the stability of magnetic multi-core particles in physiological pH environments via electrosteric stabilization. These approaches facilitate the application of magnetic particles and MPI tracers in clinical practices such as hemorrhagic stroke diagnosis [34].

## 3. Nanotechnology Facilitating Hemorrhagic Stroke Treatment

**Figure 3:**
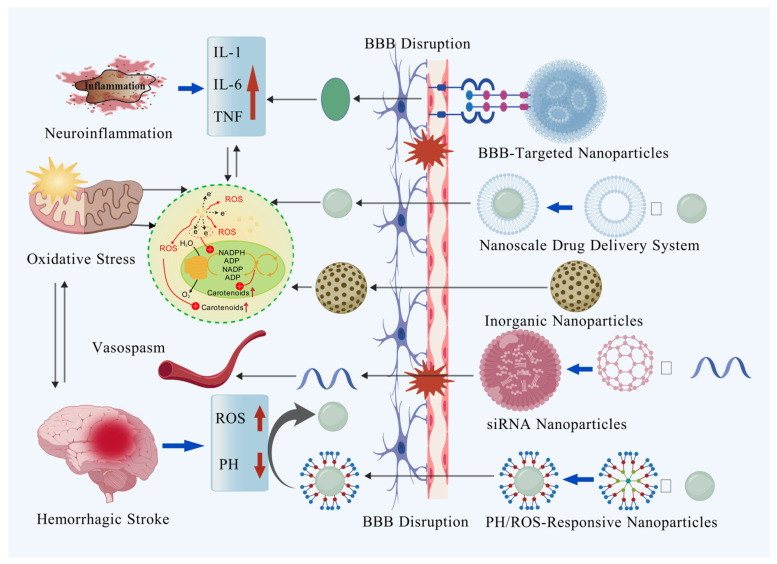
Synergistic therapeutic approach of BBB-targeted and microenvironment-responsive nanoparticles for hemorrhagic stroke.

Nanomaterials have emerged as a transformative force in addressing the multifaceted challenges of hemorrhagic stroke treatment, offering precise and versatile solutions to mitigate injury and promote recovery. During the critical acute phase requiring rapid intervention, nanocarriers constructed from biocompatible materials—such as liposomes, polymeric nanoparticles, and various inorganic nanoparticles—can penetrate the compromised BBB through tailored surface modifications like targeting peptides or antibodies. This enables site-specific delivery of therapeutic agents or functional nanoparticles, subsequently improving prognosis by intervening in multiple pathways including neuroinflammation and oxidative stress (Figure 3). Despite persistent challenges in scaling up production, ensuring long-term biocompatibility, and validating safety profiles, nanotechnology’s unique capacity to integrate targeted therapy, biomolecular regulation, and tissue repair holds immense promise for revolutionizing hemorrhagic stroke management. This advancement may ultimately steer treatment paradigms toward more personalized and efficient approaches.

### 3.1. Application of Nanodelivery Systems in the Treatment of Hemorrhagic Stroke Complications

After hemorrhagic stroke, a large amount of blood enters brain tissue, causing cerebral edema and increased intracranial pressure. This leads to mechanical compression and destruction of local tissues, reduces cortical cerebral blood flow, and causes direct damage and necrosis of surrounding neurons and glial cells. Vasospasm following SAH causes cerebrovascular constriction, resulting in insufficient cerebral blood supply and ischemic brain injury or even ischemic stroke [35]. Additionally, vasospasm affects the recurrence of cerebral aneurysms and the risk of rebleeding after SAH [36]. Nimodipine is currently recommended as a first-line treatment in clinical guidelines. However, its poor water solubility and low bioavailability limit its efficacy. In clinical practice, the standard oral administration regimen requires oral administration of 60 mg nimodipine every 4 h for 21 days [37]. This high-frequency dosing reflects nimodipine’s low oral bioavailability and significant hepatic first-pass metabolism. To improve bioavailability, intravenous administration serves as an alternative to oral delivery. However, nimodipine has extremely low solubility, so clinical intravenous formulations require approximately 40% solvent mixtures (such as ethanol and PEG400) to achieve the desired concentration. This formulation not only necessitates complex equipment (e.g., infusion pumps) and prolonged administration (approximately 10 h) but may also cause adverse reactions such as patient pain, local irritation, and even phlebitis [38]. Given the limitations of existing formulations, researchers are developing more efficient nimodipine formulations and nanodelivery systems to improve solubility, bioavailability, and patient compliance, aiming to provide better treatment options for SAH patients. Yang et al. [39] first constructed nimodipine-loaded liposomes using materials such as phosphatidylcholine, cholesterol, poloxamer 188, and sodium deoxycholate, achieving an encapsulation efficiency of 89.9% and a particle size of 200 nm. Pharmacokinetic studies demonstrated that compared to commercial formulations, this liposome showed significant advantages after intravenous administration in New Zealand rabbits: increased peak plasma concentration, prolonged elimination half-life, expanded area under the curve, and significantly extended mean residence time in plasma. Xiong et al. [38] dispersed nimodipine powder in an aqueous surfactant solution containing poloxamer 188, sodium cholate, and mannitol, successfully preparing a nimodipine nanosuspension. Research found that compared to nimodipine ethanol solution, this nanosuspension significantly reduced the incidence of vascular irritation symptoms and exhibited good tolerance with no obvious adverse side effects observed. With the deepening of research, Zech et al. [40] prepared nimodipine-loaded poly lactic-co-glycolic acid (PLGA) nanofibers using electrospinning technology. In vitro studies showed that these nanofibers were non-cytotoxic and could significantly reduce cell death under stress conditions; however, relevant in vivo experimental studies have not yet been conducted, and the specific implantation method requires further investigation. Huang et al. [41] developed a nimodipine-loaded nanoemulsion with a drug loading capacity 5-fold higher than traditional injections, demonstrating good stability and lower vascular irritation. Basalious et al. [42] designed nimodipine nanomicelles based on Pluronic/phosphatidylcholine/polysorbate 80 mixed micelles (PPPMMs), which achieved relative bioavailability in rat plasma and brain tissues reaching 232% and 208% of the oral solution, respectively. Additionally, a nanocarrier system based on A2B type miktoarm polymers (A = polyethylene glycol (PEG); B = polycaprolactone (PCL)) increased the water solubility of nimodipine by approximately 200-fold [43].

The BBB is one of the primary obstacles limiting the bioavailability of nimodipine and its delivery to brain tissue. To address this, researchers have developed a novel lactoferrin-modified long-circulating nanostructured lipid carrier (Lf-NLC), which features small particle size, uniform distribution, and high drug loading capacity. Studies indicate that Lf-NLC can not only effectively deliver nimodipine to brain tissue through receptor-mediated endocytosis but also exhibits significant neuroprotective effects by reducing cell apoptosis [44]. Additionally, intranasal administration represents a strategy to bypass BBB restrictions and hepatic first-pass metabolism while minimizing gastrointestinal side effects. Rashed et al. [45] developed 99mTc-labeled nimodipine lipid polymer micelles (99mTc-NM-LPMs). Their findings showed that compared to intravenous injection of 99mTc-NM solution, the LPM drug delivery system significantly reduced cardiac radioactivity and cardiovascular adverse reactions. Furthermore, intranasal administration of 99mTc-NM-LPMs achieved a notably higher brain drug concentration and brain/blood drug concentration ratio compared to intravenous administration.

Similarly, Mohsen et al. [46] also demonstrated that compared to intravenous injection, intranasal administration of nimodipine-loaded lipid nanocapsules (LNCs) significantly prolonged the mean residence time of the drug and increased drug concentration in brain tissue. Drug detection in the brain was possible as early as 5 min after administration, and drug concentrations remained higher than those achieved via intravenous administration at all sampled time points. Additionally, it is worth noting that leveraging the thermosensitive properties and mucoadhesive characteristics of poloxamer, researchers developed an in situ gel nasal delivery system loaded with nimodipine liposomes. This system not only inhibits nasal ciliary clearance and enhances drug absorption but also effectively crosses the BBB through nasal administration. Rat experiments confirmed its significant brain-targeting capability [47]. Although nanocarriers with sustained drug release are increasingly used to overcome the limitations of nimodipine’s low bioavailability and solubility, this approach only aids continuous drug release during the peak period of vasospasm development rather than on-demand release. Therefore, Döring et al. [48] utilized the chick chorioallantoic membrane model to investigate an ultrasound-mediated nimodipine nanodrug release system using Pluronic^®^ F-127 block copolymers as nanocarriers. Results showed that after inducing vasospasm, the average blood vessel diameter was only 57% of the normal state; however, after ultrasound-triggered drug release, the vessel diameter significantly recovered to 89% of the normal state. Nevertheless, to date, the effectiveness of this system has not been validated in in vivo models of post-SAH vasospasm.

Additionally, studies indicate that ropivacaine nanoparticles hold potential application value in treating vasospasm following SAH. Bai et al. [49] developed ropivacaine-loaded liposomes and demonstrated in a New Zealand white rabbit SAH model that, compared to the ropivacaine group, the drug-loaded group significantly reduced basilar artery blood flow velocity, neural injury markers, and endothelin-1 levels, while simultaneously increasing vessel diameter and endothelial nitric oxide synthase (eNOS) expression, reducing endothelial cell apoptosis, and alleviating vasospasm. Similarly, Sun et al. [50] prepared PLGA-ropivacaine nanoparticles using a modified acrylamide polymerization gel method, which exhibited equivalent therapeutic efficacy in a New Zealand white rabbit SAH model. Research reveals that heat shock protein 20 (HSP20) and heat shock protein 27 (HSP27) play critical roles in vascular regulation. HSP20 promotes vasodilation through cyclic nucleotide-dependent protein kinase phosphorylation, which activates cofilin and depolymerizes actin. Conversely, HSP27 maintains vascular tension by inhibiting actin depolymerization via the p38MAPK/MK2 pathway. However, the therapeutic effects of phosphorylated HSP20 peptide (p-HSP20) and MK2 inhibitory peptide (MK2i) are limited by endosomal barrier effects, requiring high doses to achieve efficacy. To address this, researchers [51] developed poly (propylacrylic acid) (PPAA)-based nanoparticles that enhance cytoplasmic delivery of HSP20 and MK2i peptides through pH-dependent endosomal escape mechanisms, significantly improving vasodilatory capacity. Furthermore, Hocking et al. [52] constructed PPAA nanoparticles loaded with HSP20 siRNA and HSP27 recombinant protein. Treatment of rat aortas with these nanoparticles reduced HSP20 expression, enhanced phenylephrine-induced vasoconstriction, and diminished sodium nitroprusside-mediated vasodilation. However, further validation in SAH-related vasospasm models remains necessary.

### 3.2. Multiple Applications and Breakthroughs of the Nanometer Drug Delivery System in the Treatment of Hemorrhagic Stroke

Nanomaterials have demonstrated broad application prospects in drug loading and regulating the local microenvironment for the treatment of hemorrhagic stroke. Graphene, as a novel carbon nanomaterial, can serve as a drug carrier with high loading capacity due to its unique physicochemical properties. Research indicates that functionalized graphene oxide nanosheets (FGO) have been demonstrated to be biocompatible coatings which can significantly enhance drug loading efficiency and stability while exhibiting no cytotoxicity. Based on this, researchers [53] loaded transcriptional activator peptide and polyethylene glycol-modified graphene oxide nanosheets with pirfenidone for the treatment of SAH. Results showed that the efficient release of pirfenidone–FGO significantly alleviated neuroinflammation following SAH. Additionally, FGO exhibits excellent near-infrared absorption properties, which can be utilized for photoacoustic imaging to achieve rapid real-time monitoring of brain tissue after SAH. In another study, researchers prepared rosuvastatin-loaded nanomicelles using PEG-PCL copolymer as a carrier via the co-solvent evaporation method. In vitro experiments confirmed their good biosafety, while in vivo studies demonstrated that compared with the carrier group and rosuvastatin group, the rosuvastatin-loaded nanomicelles significantly promoted the polarization of microglia/macrophages toward the M2 phenotype, inhibited inflammatory cell infiltration, reduced pro-inflammatory cytokine levels, and upregulated the expression of the anti-inflammatory cytokine IL-10. This resulted in reduced neuronal degeneration, alleviated brain edema, and improved neurological deficit [54]. Furthermore, researchers have designed rosuvastatin-loaded human H-ferritin nanoparticles (Rsv@HFn) as a brain-targeted nano-platform. This nano-platform enhances the ability of the drug to cross the BBB increases its accumulation at the injury site, and improves its therapeutic effect. Rsv@HFn also promotes the translocation of Nrf-2 to the cell nucleus, increases the expression of HO-1 and CD91, facilitates the shift of M1 microglia to the M2 phenotype, and reduces neuroinflammation and oxidative stress. In addition, Rsv@HFn improves the integrity of the BBB in ICH mice, reduces cerebral edema, and alleviates neuropathological damage [55]. Similarly, Gong et al. [56] developed pH-sensitive liposomes (PSLs) encapsulating fingolimod (FTY720) and ammonia borane (AB) (PSL-FTY720/AB). Research indicated that compared with the cerebral hemorrhage group, FTY720 group, and PSL-FTY720 group, the PSL-FTY720/AB treatment group exhibited the best efficacy in reducing brain edema in the ipsilateral basal ganglia and cortex, protecting BBB integrity, inhibiting neuronal apoptosis, alleviating oxidative stress and neuroinflammation, and improving neurological deficit.

Phytogenic drugs (such as curcumin, astaxanthin, quercetin, ginsenoside Rb1, and epigallocatechin-3-gallate) have garnered significant attention due to their robust antioxidant and anti-inflammatory properties. However, their clinical applications are constrained by low bioavailability resulting from poor stability, solubility, and BBB permeability. Nanodrug delivery systems offer effective strategies to address these challenges. For instance, researchers synthesized ginsenoside Rb1 carbon quantum dots (RBCQDs) with a size range of 6–8 nm. Compared to free ginsenoside Rb1, intrathecal injection of Cy5-labeled RBCQDs significantly enhanced drug accumulation in brain tissue. Through mechanisms such as reducing oxidative stress, inhibiting ferroptosis, and neuronal apoptosis, RBCQDs decreased brain edema and brain tissue water content, restored blood perfusion in the dura mater region, and improved motor neurological function in mice [57]. Furthermore, PEGylated-PLGA-based epigallocatechin-3-gallate nanoparticles also exhibit remarkable sustained-release properties [58]. Quercetin, a polyphenolic compound, exhibits in vitro antioxidant activity, but its high lipophilicity leads to low bioavailability in vivo. The quercetin-loaded nanoemulsion developed by Galho et al. [59] significantly reduced hematoma volume in ICH rats, maintained glutathione S-transferase activity, and increased glutathione content and total antioxidant capacity. Astaxanthin (ATX), a carotenoid, can alleviate cerebral oxidative stress injury, inflammatory responses, and apoptosis by activating antioxidant pathways. Researchers constructed Fe_3_O_4_-based transferrin-conjugated PEG-encapsulated ATX nanoparticles. These nanoparticles effectively internalized into the cytoplasm of primary neurons within 6 h and upregulated Bcl-2 expression while downregulating Bax and caspase-3 expression 12 h after oxyhemoglobin exposure [60]. Furthermore, researchers used catechin from tea as the raw material to prepare thiol-terminated PEG-modified polyphenol nanomedicine through oxidative polymerization and self-assembly. With its strong antioxidant and metal-chelating properties, this nanomedicine can maintain the integrity of the BBB, reduce cerebral edema, significantly improve the survival rate of mice with ICH, and alleviate their neurological deficits. Its mechanism of action involves chelating iron ions, enhancing the antioxidant capacity of tissues, reducing oxidative stress, and inhibiting iron deposition, which is expected to become a targeted strategy for the treatment of ICH and other iron overload-related diseases [61]. Notably, Cai et al. [62] developed a stimulus-responsive nanocarrier by encapsulating the ultrasonic cavitation agent perfluorocarbon (PFH), ATX, and fluorescent dye IR780 with polydopamine (PDA) to construct ultrasound-triggered release nanoparticles (AUT NPs). These nanoparticles exhibited high stability and good biosafety in physiological environments. Upon ultrasound stimulation, they responded to PFH phase transition-induced shell breach, destructing the nanostructure in situ and enabling targeted drug release to neurons, thereby exerting antioxidant and anti-apoptotic effects.

Curcumin, a natural compound extracted from turmeric rhizomes, demonstrates significant antioxidant and anti-inflammatory activities. To enhance its bioavailability, researchers have developed various curcumin nanodelivery systems, including curcumin nanoemulsion [63], polymer-based nanoparticles [64,65,66], and mPEG-PCL nanoparticles [67]. Compared with free curcumin, these nanoparticles significantly increased drug concentration in mouse brains and exhibited stronger neuroprotective effects in inhibiting iron deposition around hematomas, reducing ROS generation, alleviating BBB disruption, decreasing brain edema, and inhibiting neuronal apoptosis, thus effectively improving neurological function [63,64,65,66,67]. Additionally, Duan et al. [67] constructed a curcumin nanodrug delivery system using mPEG-PCL encapsulation. By leveraging the advantages of intranasal administration, they further validated in vitro and in vivo ICH models that curcumin exerts neuroprotection by regulating neuroinflammation and reducing neuronal inflammatory death.

### 3.3. The Intervention Efficacy of Inorganic Nanoparticles and Nanozymes in Hemorrhagic Stroke

In the field of biomedical research, inorganic nanoparticles, nanozymes, and cerium nanoparticles (CeNPs) have demonstrated remarkable application potential in eliminating inflammation and oxidative stress. For example, there exists a dynamic equilibrium between the +3 and +4 valence states of cerium ions on the surface of cerium dioxide. This characteristic enables it to mimic the functions of superoxide dismutase and catalase. In an oxidative stress environment, CeNPs can effectively scavenge superoxide anions and hydrogen peroxide through the Ce^3+^/Ce^4+^ redox cycle, converting them into oxygen and water, thus reducing oxidative stress damage. At the same time, CeNPs can also regulate the expression of inflammatory cytokines, inhibit the cascade amplification of the inflammatory response, and reduce the damage of inflammation to neurons, showing great potential in biomedical applications [68]. Kang et al. [69] coated the synthesized cerium dioxide nanoparticles with phospholipid-PEG, which significantly improved their biocompatibility. In vitro experiments showed that, compared with the control group, CeNPs could reduce the level of oxidative stress induced by hematin, decrease the content of nitrite formed from NO, alleviate cytotoxicity, and relieve the inflammatory response. In vivo studies showed that, after intravenous administration, CeNPs mainly accumulated in the hemorrhagic hemisphere. They could not only effectively reduce brain edema but also inhibit the recruitment of microglia/macrophages around the hemorrhagic lesion and the expression of inflammatory proteins. Similarly, Jeong et al. [70] found in a rodent SAH model study that although the severity of hemorrhage in the cerium dioxide nanoparticle group was comparable to that in the saline group, cerium dioxide nanoparticles significantly reduced neuronal death, macrophage infiltration, and brain edema after SAH, effectively improving mouse survival rate and neurological functional outcomes. Moreover, CeNPs were more abundantly distributed in the ipsilateral hemisphere of arterial rupture rather than the contralateral hemisphere. Moreover, Cha et al. [71] pioneered the development of lipid bilayer-coated magnetic mesoporous silica nanoparticles doped with CeNPs and MRI contrast-enhancing iron oxide particles (LMCs). The study demonstrated that intracerebrally injected LMCs could directly reach the perihematomal region and be phagocytosed by macrophages, significantly alleviating cerebral edema by reducing inflammatory macrophage infiltration, while also being clearly visualized in cerebral magnetic resonance imaging. Perfluorooctylbromide (PFOB), as a new-generation perfluorocarbon (PFC), has been widely applied in biomedical fields due to its exceptional oxygen-carrying capacity, excellent diffusion capability, low surface tension, and high gas solubility. Research indicated that, compared to the SAH model group, PFOB nanoparticles markedly reduced the severity of cerebral edema, decreased neuronal apoptosis, suppressed Caspase-3 activation and Bax expression, and upregulated Bcl-2 expression [72]. The study by Xu et al. [73] similarly found that PFOB nanoparticles could significantly reduce cerebral water content, decrease Evans blue extravasation, and lower the proportion of neuronal apoptosis in the hippocampal region in a dose-dependent manner by suppressing the expression of HIF-1α, VEGF, and BNIP3. PEG-conjugated hydrophilic carbon clusters (PEG-HCC), which are graphene-nanobased, have a large number of oxygen-containing functional groups introduced via oxidation, and are covalently conjugated with PEG to form PEG-HCC with good hydrophilicity and biocompatibility, have demonstrated therapeutic effects in models of ischemic stroke and traumatic brain injury. However, research indicates that while PEG-HCC inhibits heme-induced cellular senescence, it sensitizes cells to iron-mediated toxicity and increases susceptibility to ferroptosis. To address this, Dharmalingam et al. [74] successfully developed multifunctional nanoparticles (DEF-HCC–PEG) by covalently linking deferoxamine (DEF) to PEG-HCCs. In vitro experiments revealed that, compared to treatment with PEG-HCC or DEF alone, DEF-HCC-PEG treatment restored the viability of heme-treated cells with significantly higher efficiency, markedly reduced levels of DNA damage markers γH2AX and p-53BP1, effectively prevented heme-induced plasmid DNA strand breaks in vitro, and, unlike PEG-HCC, exhibited the ability to suppress cellular sensitivity to ferroptosis. In vivo studies further demonstrated that DEF-HCC-PEG treatment significantly restored the integrity of nuclear and mitochondrial genomes and substantially downregulated senescence-associated factors, including ANKRD1, EDN1, p21, and PVRL4. Furthermore, researchers [75] performed hematoma aspiration in ICH rats and injected a self-assembling peptide nanofiber scaffold (SAPNS) into the hemorrhagic cavity to ensure close contact with brain tissue. The results revealed that SAPNS significantly reduced pseudocavity formation, inhibited apoptosis, and improved the recovery of sensorimotor functions.

### 3.4. Nanotechnology-Based Targeted Therapy for Molecular Pathway Gene Expression Post-ICH

In the treatment of ICH, gene therapies targeting molecular pathways (e.g., siRNA and antibodies) show promise, yet their clinical translation is hindered by challenges such as poor circulatory stability, low bioavailability, and off-target effects. In recent years, nanotechnology has provided novel strategies to address these limitations. Tetrahedral framework nucleic acids (tFNAs), a class of artificial nucleic acid-based nanostructures, have emerged as a major research focus due to their three-dimensional polyhedral structure and broad biological functionalities. Chen et al. [76] investigated the therapeutic efficacy of tFNAs in mitigating diffuse microvascular endothelial cell injury following SAH. In a heme-induced cytotoxicity model, tFNAs alleviated heme-mediated damage, inhibited apoptosis, and promoted angiogenesis. In vivo studies further demonstrated that tFNAs administered locally attenuated SAH-induced injury, restored the population of brain microvascular endothelial cells (BMECs) in the endothelial layer, strengthened intercellular junctions, and improved neurological outcomes. JetPEI (Jet Polyethylenimine) nanoparticles, a PEI-based gene transfection reagent, form complexes with DNA or RNA to enhance nucleic acid uptake efficiency and promote gene transfection. As a cationic polymer, PEI binds negatively charged nucleic acids to form nanoparticles, improving intracellular transport. Researchers demonstrated that tail vein injection of miR-195-loaded JetPEI nanoparticles significantly reduced cerebral edema, lesion volume, and BBB leakage in ICH mice, alongside improved neurological scores [77]. The Tat peptide-decorated gelatin-siloxane (Tat-GS) nanoparticle, a biocompatible carrier, exhibits superior gene transfection efficiency compared to commercial reagents. Calcitonin gene-related peptide (CGRP), a potent vasodilator of arteries and veins, has shown promise in preventing post-SAH vasospasm. Researchers [78] investigated Tat-GS nanoparticles loaded with the CGRP gene (pLXSN-CGRP) for improving neurological outcomes and alleviating vasospasm in SAH models. Results revealed that Tat-GS nanoparticles achieved 1.71-fold higher sustained CGRP expression in endothelial cells compared to unmodified gelatin-siloxane nanoparticles and 6.92-fold higher than naked pLXSN-CGRP. In vivo studies further confirmed that Tat-GS/pLXSN-CGRP treatment yielded better neurological recovery and reduced vasospasm severity. The RGD-containing elastin-like polypeptide (REP) is a synthetic peptide incorporating the natural adhesion motif RGD (Arg-Gly-Asp). Studies show that REP administration reduces hematoma volume, decreases activated microglial cell counts, downregulates vWF expression, and inhibits IgG leakage into brain parenchyma following ICH [79]. Mo et al. [80] further encapsulated REP with mPEG-PLGA to develop REP nanoparticles (REP-NPs), which exhibit enhanced oral absorption and sustained drug release in vivo. Experimental results demonstrated that, compared to ICH and REP-only groups, REP-NP treatment significantly reduced hematoma volume, attenuated neuronal degeneration and iron overload deposition, and improved motor coordination in ICH mice. Similarly, the anti-transferrin receptor monoclonal antibody OX26 exerts neuroprotective effects by inhibiting the JAK2/STAT3 signaling pathway. Researchers constructed OX26-conjugated PEGylated selenium nanoparticles (OX26-PEG-Se) and found that epidural injection of these nanoparticles in SAH models significantly reduced serum levels of inflammatory markers (NSE and S100B) and the vasoconstrictor endothelin-1 (ET1), while upregulating the vasodilator eNOS. This intervention improved motor function and reduced neurological injury risk [81]. Iron oxide magnetic nanoparticles (MNPs), characterized by their ultra-small size, strong magnetism, and stable physicochemical properties, hold significant potential in biomedical applications. Magnetic targeting, a non-invasive drug delivery strategy, optimizes spatial drug distribution, while focused ultrasound (FUS) temporarily enhances BBB permeability through synergistic interaction with microbubbles. Studies indicate that 15-deoxy-Δ12,14-prostaglandin J2 (15d-PGJ2), a physiological agonist of peroxisome proliferator-activated receptor gamma (PPARγ), promotes macrophage-mediated phagocytosis of hematomas and necrotic cells by activating platelet glycoprotein 4, thereby alleviating cerebral edema, suppressing neuroinflammation, and exerting neuroprotective effects. Wang et al. [82] pioneered the loading of Cy5.5-labeled 15d-PGJ2 onto MNPs and innovatively combined magnetic targeting with FUS for intracranial drug delivery, confirming the system’s safety. In vivo experiments demonstrated that magnetic targeting effectively concentrated 15d-PGJ2-MNPs in the brains of ICH mice, with FUS further ensuring stable and uniform drug distribution. Mechanistic studies revealed that dispersed 15d-PGJ2-MNPs more efficiently activated PPARγ, enhancing microglial phagocytic capacity toward hematomas. Imaging and histological analyses showed that the 15d-PGJ2-MNPs + magnetic targeting + FUS treatment group exhibited significantly reduced hematoma volume by day 3 and near-complete clearance by day 7, alongside superior restoration of perihematomal brain tissue morphology compared to the ICH control group. Additionally, phosphatidylserine-presenting liposomes, valued for their macrophage-targeting ability, have been explored as drug carriers. Han et al. [83] developed an interleukin-10 (IL-10)-loaded phosphatidylserine liposome nanoparticle system (PSL-IL10) and evaluated its efficacy via intranasal administration in a collagenase-induced ICH mouse model. Results demonstrated that PSL-IL10 markedly suppressed glial cell activation, enhanced microglial/macrophage phagocytic function, accelerated hematoma resolution, and improved neurological outcomes.

### 3.5. Nanotechnology-Driven Development of Transplantation Stem Cell Therapy

Stem cell transplantation therapy promotes neural repair by leveraging the self-renewal and differentiation capabilities of stem cells, with clinical studies confirming its ability to significantly improve patients’ neurological function and quality of life. However, the complex microenvironment at hemorrhagic sites (e.g., iron overload, hypoxia, and inflammatory responses) severely compromises the survival and functionality of transplanted stem cells, posing a critical bottleneck for therapeutic efficacy [84]. To address this challenge, Gong et al. [85] developed a core–shell hydrogel system using a rapidly degradable low-molecular-weight keratin hydrogel as the outer shell to scavenge iron overload and a high-molecular-weight keratin hydrogel embedded with epidermal growth factor (EGF)- and fibroblast growth factor (FGF)-loaded PLGA nanoparticles as the inner core to support bone marrow mesenchymal stem cell (BMSC) growth. Experiments demonstrated that this system not only effectively reduced intracellular iron burden, protected BMSCs from hemoglobin toxicity, and alleviated cerebral edema and atrophy by clearing hematoma iron deposits but also promoted BMSC differentiation into neurons, ultimately improving limb dysfunction. Concurrently, Chen et al. [86] modified human umbilical cord mesenchymal stem cells (hUC-MSCs) with MRI-trackable iron oxide magnetic nanoparticles (Ir-MNAs). They found that Ir-MNA-coated stem cells selectively accumulated in cerebral hemorrhage foci, enhancing neuroprotection by suppressing neuroinflammation, mitigating oxidative stress, and reducing mitochondrial damage. Notably, fractionated dosing (administered at 3 h and 24 h post-ICH) achieved superior neurological recovery compared to single-dose therapy. Although these advances are encouraging, fully realizing the potential of stem cell-based therapies for neurological disorders still requires addressing challenges such as optimizing stem cell sources, differentiation protocols, and delivery methods to ensure safety, efficacy, and reproducibility, as well as carefully evaluating and long-term monitoring potential post-transplant risks like tumorigenicity and immune rejection [87].

### 3.6. The Current Limitations of Nanomaterials

Although nanomaterials show great promise in treating hemorrhagic stroke, their application still faces several significant limitations. At the manufacturing level, the complex production processes and high costs of nanomaterials pose major barriers to large-scale adoption. Current synthesis methods heavily rely on advanced equipment and technology, rendering production both intricate and expensive. This complexity severely hampers the scalability and widespread clinical use of nanomedicines. It also challenges the reproducibility of traditional batch synthesis methods on an industrial scale, limiting their feasibility for broad clinical application [88,89]. Additionally, variability in nanoparticle size, surface functionalization, or encapsulation efficiency can significantly impact therapeutic outcomes. Toxicological challenges remain equally prominent. Preclinical toxicology issues are substantial, with nanomedicine-related toxic effects categorized into seven types based on underlying mechanisms: cytotoxicity, immunotoxicity, and nanotoxicity, among others [90]. Moreover, long-term nonspecific accumulation of nanomaterials in tissues may induce toxicity. Inorganic nanoparticles like iron oxide and quantum dots risk accumulating in sensitive tissues, potentially triggering oxidative stress and chronic inflammation [91]. Even nanoparticles with good biocompatibility may provoke unintended immune responses or disrupt the homeostasis of the central nervous system microenvironment—all factors impeding large-scale clinical translation. Furthermore, when translating nanoparticle-based therapies for central nervous system diseases to clinical use, ensuring long-term safety remains a critical challenge [92]. The interactions between nanoparticles and neural or systemic environments are not yet fully understood, which also restricts their broader implementation in clinical settings.

## 4. Conclusions and Perspectives

### 4.1. Conclusions

Hemorrhagic stroke, a life-threatening neurological disorder, imposes a heavy societal and familial burden due to its high rates of disability and mortality. Conventional therapies are limited by the BBB and inefficient drug delivery, hindering effective intervention in its complex pathological progression. This review systematically integrates research advances in nanotechnology for hemorrhagic stroke diagnosis and treatment, and for the first time constructs a complete “diagnosis–delivery–treatment” framework, clearly demonstrating the innovative progress and application potential of this field (Table 1). Nanotechnology, leveraging its unique physicochemical properties and multifunctional integration, has provided breakthrough solutions for precise hemorrhagic stroke management. In diagnostics, magnetic nanoparticle-enhanced MPI enables bedside real-time monitoring of hematoma evolution and cerebral perfusion, surpassing traditional imaging methods in temporal resolution and sensitivity, thus offering critical insights for clinical decision-making. Therapeutically, nanodrug delivery systems significantly enhance the brain targeting and bioavailability of neuroprotective agents, plant-derived drugs, and gene therapy vectors by improving drug solubility, traversing the BBB, and enabling responsive release. Multifunctional inorganic nanomaterials, such as CeO_2_, graphene, and PFOB nanoparticles, mitigate cerebral edema and neuronal damage through antioxidant, anti-inflammatory, and free radical-scavenging mechanisms. Additionally, nanocarrier-mediated gene delivery and stem cell transplantation strategies have opened new avenues for modulating molecular pathways and promoting neural repair.

### 4.2. Future Perspectives

Despite the immense potential of nanotechnology in hemorrhagic stroke management, its clinical translation still faces substantial challenges, including comprehensive biosafety evaluation, formulation standardization for batch-to-batch consistency, and rigorous validation of long-term therapeutic efficacy in large animal models. Future research efforts should focus on three key directions: first, optimizing nanomaterial design to enhance biocompatibility and reduce off-target effects, such as developing biodegradable polymers or cell membrane-camouflaged nanoparticles; second, advancing intelligent stimulus-responsive delivery systems that can precisely respond to hemorrhagic stroke-specific microenvironmental cues for spatiotemporally controlled drug release; third, integrating multimodal therapeutic strategies to synergistically intervene in the complex pathological cascade of hemorrhagic stroke. Moreover, cross-disciplinary collaboration holds great promise.

## Figures and Tables

**Table 1:** Multiple applications of nanotechnology in the diagnosis and treatment of hemorrhagic stroke.

Nanomaterial	Disease	Main Research Conclusions	References
Indium and Dil double-labeled lipid nanoparticles	ICH	After intravenous injection, brain liposomes show a biphasic accumulation peak, with the most accumulation in the hematoma area and co-localization with activated microglia. Mechanistic studies find that in the early ICH stage, Caveolin-1-mediated transcytosis is enhanced, and in the later stage, Claudin-5 is significantly downregulated, revealing a dual-regulatory BBB permeability.	[28]
VCAM-SOD-lipid nanoparticles	ICH	The brain uptake of VCAM-SOD-lipid nanoparticles is significantly higher than that of SOD-lipid nanoparticles. And, the delivery efficiency of the targeted liposomes remains stable after the acute phase, while the delivery efficiency of the passive leakage pathway decays rapidly.	[29]
Superparamagnetic iron oxide nanoparticle-Synomag-D	ICH	Intravenous injection of superparamagnetic iron oxide nanoparticle-Synomag-D enables bedside detection within 3 min using MPI technology. MPI can clearly distinguish liquid and coagulated hematoma areas, monitor cerebral perfusion status synchronously, providing a key basis for choosing surgical timing. Moreover, it can sensitively identify complications like increased intracranial pressure and vasospasm.	[33]
Qdot^®^ 655 nanocrystal	SAH	After injecting Qdot^®^ 655 nanocrystals, it was found that within 1 min after SAH, the capillary network ruptured, and the blood flow velocity in the pre-arterioles decreased by 85%.	[30]
Nimodipine–lipid nanoparticles; Nimodipine nano-suspension; Nimodipine–PLGA nanofibers; Nimodipine nanoemulsion; PPPMM; Nimodipine–A2B type miktoarm polymers; lactoferrin modified long circulation nanostructured lipid carriers; 99mTc-Nimodipine-LPM; Nimodipine–lipid nanocapsules; Nimodipine-Pluronic^®^ F-127	SAH	These nano-formulations have improved the solubility, bioavailability, degree of vascular irritation, and concentration in the brain of nimodipine.	[38,39,40,41,42,43,44,45,46,47,48]
Ropivacaine–lipid nanoparticles; PLGA–ropivacaine nanoparticles	SAH	Ropivacaine nanoparticles significantly reduced the blood flow velocity of the basilar artery, the levels of nerve injury markers and endothelin-1. Meanwhile, they increased the diameter of the blood vessel and the expression of endothelial nitric oxide synthase, decreased the apoptosis of endothelial cells, and alleviated vasospasm.	[49,50]
HSP20 and MK2 inhibitory peptide–PPAA nanoparticles; HSP20 siRNA and HSP27 recombinant protein–PPAA nanoparticles	SAH	Nanoparticles of PPAA loaded with HSP20 and MK2 inhibitory peptides, as well as PPAA nanoparticles of HSP20 siRNA and HSP27 recombinant protein, promote the cytoplasmic delivery of HSP20, MK2 inhibitory peptides, HSP20 siRNA, and HSP27 recombinant protein through a pH-dependent endosomal escape mechanism, significantly enhancing the vasodilatory ability.	[51,52]
Pirfenidone–functionalized nanoscale graphene oxide nanoparticles	SAH	The efficient release of pirfenidone–FGO significantly alleviated the neuroinflammation after SAH. In addition, pirfenidone–FGO also exhibited excellent near-infrared absorption properties and could be used for photoacoustic imaging to achieve rapid and real-time monitoring of brain tissue after SAH.	[53]
Rosuvastatin-PEG-PCL nanomicelles	ICH	The rosuvastatin-loaded nanomicelles significantly promoted the polarization of microglia/macrophages towards the M2 phenotype, inhibited the infiltration of inflammatory cells, reduced the levels of pro-inflammatory factors, and upregulated the expression of the anti-inflammatory factor IL-10, thereby reducing neuronal degeneration, alleviating cerebral edema, and improving neurological deficits.	[54]
Rsv@HFn	ICH	This nano-platform enhances the ability of the drug to cross the BBB, increases its accumulation at the injury site, and improves its therapeutic effect. Rsv@HFn also promotes the translocation of Nrf-2 to the cell nucleus, increases the expression of HO-1 and CD91, facilitates the shift of M1 microglia to the M2 phenotype, and reduces neuroinflammation and oxidative stress. In addition, Rsv@HFn improves the integrity of the BBB in ICH mice, reduces cerebral edema, and alleviates neuropathological damage.	[55]
PSL-FTY720/AB	ICH	Compared with the ICH group, the FTY720 group, and the PSL-FTY720 group, the PSL-FTY720/AB treatment group showed the best therapeutic effects in reducing cerebral edema in the ipsilateral basal ganglia and cerebral cortex, protecting the integrity of the BBB, inhibiting neuronal apoptosis, alleviating oxidative stress and neuroinflammation, and improving neurological deficits.	[56]
RBCQDs	ICH	Compared with free ginsenoside Rb1, Cy5-labeled RBCQDs significantly increased the drug accumulation in brain tissue. Through mechanisms such as alleviating oxidative stress and inhibiting ferroptosis and neuronal apoptosis, it reduced cerebral edema and the water content in brain tissue, restored blood perfusion in the dura mater area, and improved the motor nerve function of mice.	[57]
PEGylated-PLGA EGCG nanoparticles	ICH	PEGylated-PLGA EGCG nanoparticles have remarkable sustained-release characteristics.	[58]
Quercetin-loaded nanoemulsion	ICH	Quercetin-loaded nanoemulsion significantly reduced the hematoma volume in rats with ICH, maintained the activity of glutathione S-transferase, and increased the content of glutathione and the total antioxidant capacity.	[59]
Transferrin conjugated to PEG-encapsulated ATX nanoparticles	ICH	ATX nanoparticles can be effectively internalized into the cytoplasm of primary neurons within 6 h, and upregulate the expression of Bcl-2 12 h after exposure to oxyhemoglobin, while downregulating the expressions of Bax and caspase-3.	[60]
Catechin-based polyphenol nanoparticles surface-modified by thiol-terminated poly(ethylene glycol)	ICH	CNPs@PEG effectively maintained BBB integrity, reduced brain edema, significantly increased the survival rate of mice with cerebral hemorrhage and markedly improved neurological deficits after ICH. Mechanistically, CNPs@PEG accomplishes this by chelating iron, enhancing tissue antioxidant capacity, reducing oxidative stress, and inhibiting iron deposition.	[61]
AUTNPs	SAH	AUTNPs respond to the phase transition of perfluorooctyl bromide induced by ultrasound, break through the shell, in situ destroy the nanostructure, and release the drug to neurons in a targeted manner, exerting antioxidant and anti-apoptotic effects.	[62]
Curcumin nanoemulsion; curcumin polymer-based nanoparticles; curcumin–PEG-PCL nanoparticles	ICH AND ICH	Compared with free curcumin, curcumin nanoparticles significantly increased the drug concentration in the brains of mice. They also showed stronger neuroprotective effects in aspects such as inhibiting iron deposition in the brain tissue around the hematoma, reducing the generation of reactive oxygen species, alleviating the damage of the BBB, reducing the degree of cerebral edema, and inhibiting neuronal apoptosis.	[63,64,65,66,67]
Cerium dioxide nanoparticles	ICH AND ICH	Cerium nanoparticles can reduce the level of oxidative stress induced by heme, decrease the content of nitrite formed by nitric oxide, alleviate cytotoxicity, and mitigate the inflammatory response. In vivo studies have found that after intravenous administration, CeNPs mainly accumulate in the hemorrhagic hemisphere. They can not only effectively reduce cerebral edema, but also inhibit the recruitment of microglia/macrophages around the bleeding focus and the expression of inflammatory proteins. Although the severity of hemorrhage in the cerium dioxide nanoparticle group was comparable to that in the normal saline group, cerium dioxide nanoparticles significantly reduced neuronal death, macrophage infiltration, and cerebral edema after SAH, effectively improving the survival rate of mice and the prognosis of neurological function.	[69,70]
Lipid-coated magnetic mesoporous silica nanoparticles doped with cerium dioxide (LMC)	ICH	Intracerebral injection of LMC can directly reach the area around the hematoma and be phagocytosed by macrophages. By reducing the infiltration of inflammatory macrophages, it can significantly alleviate cerebral edema and can be clearly visualized in brain magnetic resonance imaging.	[71]
Perfluorooctyl-bromide nanoparticles	SAH	Compared with the SAH model group, PFOB nanoparticles can significantly reduce the degree of cerebral edema, decrease neuronal apoptosis, inhibit the activation of Caspase-3 and the expression of Bax, and increase the expression of Bcl-2. They can also inhibit the expressions of HIF-1α, VEGF, and BNIP3, significantly reduce the brain water content, decrease the extravasation of Evans blue, and lower the proportion of neuronal apoptosis in the hippocampal region in a dose-dependent manner.	[73]
DEF-HCC–PEG nanoparticles	ICH	When compared with treating cells with PEG-HCC or DEF alone, DEF-HCC-PEG nanoparticles can restore the viability of heme-treated cells with significantly higher efficiency. At the same time, they can significantly reduce the levels of DNA damage markers γH2AX and p-53BP1, effectively prevent the heme-induced plasmid DNA strand breaks in vitro. Moreover, DEF-HCC-PEG has the characteristic of inhibiting the sensitivity of cells to ferroptosis. In in vivo experiments featuring treatment with DEF-HCC-PEG nanoparticles, the integrity of the nuclear and mitochondrial genomes is significantly restored, and the expression levels of senescence-related factors ANKRD1, EDN1, p21, and PVRL4 are significantly decreased.	[74]
SAPNS	ICH	SAPNS significantly reduces the formation of the cerebral cavity after ICH, inhibits apoptosis, and improves the recovery of sensorimotor function.	[75]
tFNAs	SAH	tFNA in situ alleviated the damage caused by SAH, restored the number of BMECs and intercellular junctions in the endothelial layer, inhibited cell apoptosis, promoted angiogenesis, and improved neurological function.	[76]
JetPEI nanoparticles loaded with miR-195	ICH	JetPEI nanoparticles loaded with miR-195 significantly reduced brain edema, lesion volume, and blood–brain barrier leakage in ICH mice and improved neurological function scores.	[77]
Tat-GS nanoparticles loaded with CGRP gene	SAH	The continuous expression of CGRP in endothelial cells by Tat-GS nanoparticles encapsulating pLXSN-CGRP was 1.71 times that of gelatin-siloxane nanoparticles encapsulating pLXSN-CGRP and 6.92 times that of naked pLXSN-CGRP. Moreover, the group treated with Tat-GS nanoparticles encapsulating pLXSN-CGRP had better neurological function outcomes and less vasospasm.	[78]
REP-mPEG-PLGA nanoparticles	ICH	Compared with the ICH group and the REP group, mPEG-PLGA nanoparticles loaded with REP significantly reduced the hematoma volume after ICH, alleviated neuronal degeneration, necrosis, and iron overload deposition, and improved the motor coordination function of ICH mice.	[79]
OX26-PEG-Se nanoparticles	SAH	Compared with the control group, epidural injection of OX26-PEG-Se nanoparticles could significantly reduce the levels of serum inflammatory factors NSE and S100B and the vasoconstrictor factor ET1, and upregulate the expression of the vasodilator factor NOS, thereby significantly improving the motor function of the SAH model and reducing the risk of nerve injury.	[80]
15d-PGJ2-MNPs	ICH	Under magnetic targeting guidance, 15d-PGJ2-MNPs could be effectively enriched in the brains of ICH mice, and the combination with focused ultrasound (FUS) further achieved a stable and uniform distribution of the drug. Mechanistic studies showed that the diffused 15d-PGJ2-MNPs were more likely to activate PPARγ, thereby enhancing the phagocytic ability of microglia towards hematomas. Compared with the ICH control group, the hematoma volume in the 15d-PGJ2-MNPs + magnetic targeting + FUS treatment group was significantly reduced on the 3rd day, almost completely cleared on the 7th day, and the morphology of the brain tissue around the hematoma recovered better.	[82]
PSL-IL10 nanoparticles	ICH	PSL-IL10 significantly inhibited the activation of glial cells, enhanced the phagocytic function of microglia/macrophages, accelerated hematoma absorption, and improved neurological function.	[83]
PLGA nanoparticles loaded with EGF and FGF	ICH	Core–shell structured hydrogel system: The rapidly degradable low-molecular-weight keratin hydrogel serves as the shell to remove iron overload, while the high-molecular-weight keratin hydrogel encapsulating PLGA nanoparticles loaded with EGF and FGF acts as the core to support the growth of BMSCs. The core–shell structure can not only effectively reduce the intracellular iron load and protect BMSCs from hemoglobin toxicity, but also significantly alleviate brain edema and brain atrophy by efficiently removing iron deposits in hematomas, promote the differentiation of BMSCs into neurons, and ultimately improve limb dysfunction.	[85]
Ir-MNA-modified human umbilical cord mesenchymal stem cells	ICH	Human umbilical cord mesenchymal stem cells modified with Ir-MNA nanoparticles can be targeted and enriched in cerebral hemorrhage foci, achieving more significant brain protection effects by inhibiting neuroinflammation, reducing oxidative stress, and alleviating mitochondrial damage.	[86]

## Data Availability

Not applicable.

## Abbreviations

The following abbreviations are used in this manuscript:

AB	Ammonia Borane
ATX	Astaxanthin
BBB	Blood–Brain Barrier
BMECs	Brain Microvascular Endothelial Cells
BMSC	Bone Marrow Mesenchymal Stem Cell
CGRP	Calcitonin Gene-Related Peptide
CeNPs	Cerium Nanoparticles
DEF	Deferoxamine
EGF	Epidermal Growth Factor
ET1	Endothelin-1
FGF	Fibroblast Growth Factor
FGO	Functionalized Graphene Oxide Nanosheets
FTY720	Fingolimod
FUS	Focused Ultrasound
GS	Gelatin-Siloxane
hUC-MSCs	Human Umbilical Cord Mesenchymal Stem Cells
ICH	Intracerebral Hemorrhage
IL-10	Interleukin-10
Ir-MNAs	Iron Oxide Magnetic Nanoparticles
JAK2/STAT3	Janus Kinase 2/Signal Transducer and Activator of Transcription 3
JetPEI	Jet Polyethylenimine
Lf-NLC	Long-Circulating Nanostructured Lipid Carrier
LMCs	Lipid Bilayer-Coated Magnetic Mesoporous Silica Nanoparticles Doped with CeNPs and MRI Contrast-Enhancing Iron Oxide Particles
LNCs	Lipid Nanocapsules
MMP	Mitochondrial Membrane Potential
MNPs	Magnetic Nanoparticles
mPEG-PLGA	Methoxy Polyethylene Glycol-Poly(Lactic-Co-Glycolic Acid)
MRI	Magnetic Resonance Imaging
NSE	Neuron-Specific Enolase
OX26	Anti-Transferrin Receptor Monoclonal Antibody OX26
PDA	Polydopamine
PEI	Polyethylenimine
PEG-HCC	Poly-(Ethylene Glycol)-Conjugated Hydrophilic Carbon Clusters
PEG-PCL	Polyethylene Glycol-Poly(ε-Caprolactone)
PFC	Perfluorocarbon
PFOB	Perfluorooctylbromide
PLGA	Poly Lactic-Co-Glycolic Acid
PPARγ	Peroxisome Proliferator-Activated Receptor Gamma
PS	Phosphatidylserine
PSL	PH-Sensitive Liposomes
PSL-FTY720/AB	PH-Sensitive Liposomes Encapsulating Fingolimod and Ammonia Borane
PSL-IL10	Phosphatidylserine Liposome-Interleukin-10
pLXSN-CGRP	Plasmid LXSN Carrying CGRP Gene
RbcQDs	Rb1 Carbon Quantum Dots
REP	RGD-Containing Elastin-Like Polypeptide
REP-NPs	REP Nanoparticles
RGD	Arg-Gly-Asp
Rsv@HFn	Rosuvastatin-Loaded Human H-Ferritin Nanoparticles
SAH	Subarachnoid Hemorrhage
SAPNS	Self-Assembling Peptide Nanofiber Scaffold
S100B	S100 Calcium-Binding Protein B
siRNA	Small Interfering RNA
SPECT/CT	Single-Photon Emission Computed Tomography/Computed Tomography
Tat	Tat Peptide
Tat-GS	Tat Peptide-Decorated Gelatin-Siloxane
tFNAs	Tetrahedral Framework Nucleic Acids
15d-PGJ2	15-Deoxy-Δ12,14-Prostaglandin J2
